# The apicoplast genome of *Leucocytozoon caulleryi*, a pathogenic apicomplexan parasite of the chicken

**DOI:** 10.1007/s00436-013-3712-9

**Published:** 2013-12-04

**Authors:** Takayuki Imura, Shigeharu Sato, Yukita Sato, Daichi Sakamoto, Takashi Isobe, Koichi Murata, Anthony A. Holder, Masayoshi Yukawa

**Affiliations:** 1 Department of Veterinary Medicine, College of Bioresource Sciences, Nihon University, Fujisawa, Kanagawa 252-0880 Japan; 2 Division of Parasitology, MRC National Institute for Medical Research, London, NW7 1AA UK; 3 National Institute of Animal Health, Tsukuba, Ibaraki 305-0856 Japan; 4 Department of Animal Resource Sciences, College of Bioresource Sciences, Nihon University, Fujisawa, Kanagawa 252-0880 Japan; 5 Laboratory of Biomedical Science, Department of Veterinary Medicine, Nihon University, Fujisawa, Kanagawa 252-0880 Japan

## Abstract

*Leucocytozoon caulleryi*, a haemosporidian parasite of the chicken (*Gallus gallus domesticus*), can be highly pathogenic and often fatal. Although this parasite is extremely relevant to veterinary science, knowledge of its genomic features is limited. To gain information applicable to developing novel control methods for the parasite, we analyzed the apicoplast genome of *L. caulleryi*. This extranuclear organellar DNA of 85.1 % A + T and a unit of 34,779 bp was found to encode almost the same set of genes as the plastid genome of *Plasmodium falciparum,* including 16 tRNA and 30 protein coding genes, and except for one open reading frame, *ORF91* absent in *L. caulleryi*. As in *P. falciparum*, the *L. caulleryi* apicoplast DNA contains two sets of a unique inverted repeat (IR), each one 5,253 bp and encoding genes specifying one large and one small rRNA subunit and nine tRNAs but no protein, and separated by a unique 13 bp sequence. Studies of several haemosporidian apicoplast DNA sequences have identified a corresponding IR region; however, none of these studies has looked at the complete sequence, even for well-studied species such as *P. falciparum*. Phylogenetic studies using a concatenated amino acid sequence based on the open reading frames confirmed the close relationship between *L. caulleryi* and *Plasmodium* spp. In this study, we determined the nucleotide sequence of the entire *L. caulleryi* apicoplast genome, including the region connecting the two IR units. This is the first report of the complete nucleotide sequence of a haemosporidian apicoplast DNA with a canonical IR.

## Introduction

The phylum Apicomplexa consists of protistan parasites, including species of medical or veterinary importance such as *Plasmodium falciparum*, *Toxoplasma gondii*, and *Eimeria tenella*. One of the characteristics shared by these parasites is the presence of a vestigial plastid called the apicoplast (Foth and McFadden 2003; McFadden 2011). This plastid has been suggested to have originated from an ancient endosymbiotic alga of debated phylogenetic position (Köhler et al. 1997; Janouskovec et al. 2010). Previous studies have suggested that this organelle is involved in critical metabolic processes such as heme and isoprenoid biosynthesis (McFadden 2011; Ralph et al. 2004). Indeed, *P. falciparum* that has lost its apicoplast cannot grow unless the product of the apicoplastic isoprenoid biosynthesis, isopentenyl pyrophosphate, is supplemented to the culture medium (Yeh and DeRisi 2011). These studies have led to recognition of the apicoplast as a potential anti-*Plasmodium* drug target.

As a vestigial plastid, the apicoplast retains its own genome. To date, analysis of the apicoplast genomes of nine *Plasmodium* species, *T. gondii*, *E. tenella*, *Theileria parva*, and *Babesia bovis* has been performed (Arisue et al. 2012; Brayton et al. 2007; Cai et al. 2003; Gardner et al 2005; Lau et al. 2009; Williamson et al. 2001), and the results recorded in gene databases. These data revealed that features of the apicoplast genome are fairly well conserved between species, although they may also differ. The part of the apicoplast DNA sequence encoding the 2 rRNA genes (*rrl*, *rrs*) as well as genes for 9 tRNA species (*trnA*(UGC), *trnI*(GAU), *trnL*(UAG), *trnM*(CAU), *trnN*(GUU), *trnR*(ACG), *trnR*(UCU), *trnT*(UGU), and *trnV*(UAC)) are duplicated to form an inverted repeat (IR) in all *Plasmodium* species studied, except for the rodent malaria parasite *Plasmodium chabaudi chabaudi* (Arisue et al. 2012; Sato et al. 2013). An IR with identical features exists even in coccidian apicoplast sequences, although the corresponding sequence is not duplicated in piroplasmids. These findings suggest that the apicoplast IR was present in the common ancestor of all extant apicomplexan species and that one copy of the duplicated IR units was independently lost in the branches corresponding to piroplasmids and *P. chabaudi chabaudi* (Sato et al. 2013).

The apicomplexan genus *Leucocytozoon* consists of species which parasitize birds (Valkiunas 2005). Among them, *L. caulleryi*, which can cause fatal malaria-like symptoms in the chicken (*Gallus gallus domesticus*), is one of the species with the greatest economic impact, and it is distributed mainly throughout Asian countries including Japan (Morii et al. 1981; Morii 1992; Yu and Wang 2001). Genomic information could contribute not only to understanding the biological features of pathogens but also to the development of control procedures for infection, such as genetically recombinant vaccines or protein-targeted drugs. Trials to induce production of protective antibodies against *L. caulleryi* infection by inoculation of chickens with recombinant antigens have been reported (Ito and Gotanda 2004; Ito et al. 2005; Morii et al. 1996). We previously reported the complete mitochondrial DNA sequence as well as the structure and partial nucleotide sequence of the apicoplast DNA of *L. caulleryi* (Omori et al. 2008). However, the entire genome should reveal further understanding of this parasite’s biology. In this study, we determined the complete nucleotide sequence of the apicoplast DNA, including the region connecting the two IR units. This is the first such report of a haemosporidian IR-positive apicoplast DNA sequence.

## Materials and methods

### PCR amplification and Sanger sequence analysis of genomic DNA

Total *L. caulleryi* genomic DNA was prepared from the blood of infected chickens (designated the Niigata strain), which were obtained from a poultry farm in Niigata Prefecture, Japan. A series of PCR primers was designed to match the *Plasmodium* spp. plastid DNA sequences obtained from a genetic database. With these primers, sections of the plastid DNA were amplified from the total *L. caulleryi* genomic DNA preparation by polymerase chain reaction (PCR) and the nucleotide sequence of each PCR fragment was determined directly by Sanger sequencing as described previously (Omori et al. 2008).

### High throughput sequence analysis of genomic DNA

A library for high-throughput sequencing (HTS) was prepared from the parasite DNA using the Paired End Sample Prep Kit (Illumina) and analyzed by the Illumina method on a Genome Analyzer IIx (Illumina). From the HTS data obtained, reads matching the *trnI* gene sequence at the end of the IR unit were searched for by using BLASTN to query the NCBI database. Obtained reads were aligned using Clustal X (Larkin et al. 2007) to extend the nucleotide sequences in locations where the PCR direct sequencing method described above was insufficient.

The position and the anticodon of each tRNA gene encoded by the *L. caulleryi* plastid DNA sequence were predicted by the tRNAscan-SE server (http://lowelab.ucsc.edu/tRNAscan-SE/). All other genes were manually annotated based on homology to *P. falciparum.*

### Phylogenetic analysis

Phylogeny of the plastid genomes of *L. caulleryi* and 7 other apicomplexan species (*P. falciparum*, *P. gallinaceum*, *P. berghei*, *T. parva*, *B. bovis*, *E. tenella*, and *T. gondii*) as well as *Chromera velia*, which was chosen as the out-group, was deduced from the amino acid sequences that represent the genomes. The representative sequences, which were 2,478 residues long, were created by concatenating the amino acid sequences of the products of the following 10 genes: *rpl 2*, *rpl 6*, *rpl 14*, *rpl 16*, *rps 2*, *rps 3*, *rps 11*, *rps 12*, *tufA*, and *rpoB*. An alignment of these sequences was created using Clustal W (Larkin et al. 2007), and the phylogeny of these sequences was analyzed using MEGA5 (http://www.megasoftware.net) by the maximum likelihood method with the JTT + F + Γ model. Bootstrap analysis with 1,000 replicates was performed to estimate the confidence of the tree topology.

## Results

**Fig. 1:**
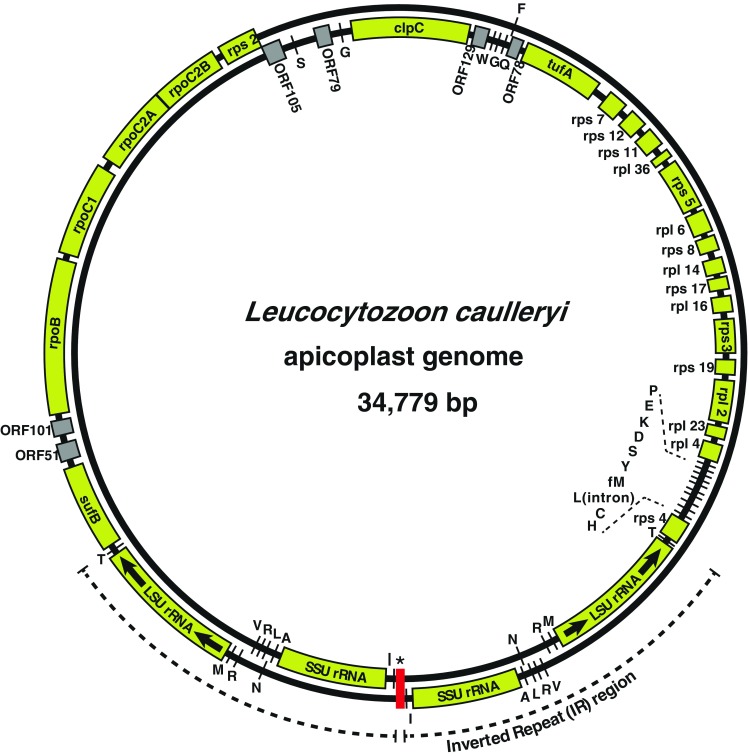
Map of the *L. caulleryi* plastid genome. *Concentric circles* represent the circular double-stranded DNA genome, and each gene annotated in the DNA sequence is illustrated on either of the two circles depending on the direction of transcription (*outer circle* clockwise; *inner circle* anticlockwise). *Yellow boxes* represent genes specifying either a protein or an rRNA, whereas *lines* and *gray boxes* indicate tRNA-coding genes or hypothetical open reading frames, respectively. Each inverted repeat (IR) unit is indicated by the *outermost dotted arcs*, and the asymmetric 13-bp sequence separating the two IR units is marked with an *asterisk*

We determined the complete nucleotide sequence of the *Leucocytozoon* plastid DNA for the first time. The *L. caulleryi* plastid DNA was 34,779 bp, and its A + T content was 85.1 % (Table 1). These values were within the range of other plastid DNA data previously reported. Like the plastid DNA of *Plasmodium* spp. and the coccidians, the plastid DNA of *L. caulleryi* had an inverted repeat (IR) that occupied about 1/3 of the unit sequence (Fig. 1). We had no problem in amplifying the majority of the plastid DNA by PCR. However, the sequence connecting the two IR units at the end closest to the tRNA-Ile gene was the exception; we had no success in obtaining PCR fragments that contained the short sequence (data not shown). It is possible that this region of the *L. caulleryi* plastid DNA forms a peculiar structure that interferes with extension of the DNA strand in PCR, similar to the “tip region” present between the two IR units of the *E. tenella* plastid DNA (Cai et al. 2003). Therefore, we analyzed the total genomic DNA extracted from the parasite preparation by whole genome sequencing with the Illumina platform, and we searched for reads corresponding to the end of the IR unit in these HTS data. These identified reads filled the gap between the two IR units, which was found to be only 13 bp, allowing us to finally obtain the complete plastid nucleotide sequence.

**Table 1:** Comparison of apicoplast genomes

Species	*Leucocytozoon caulleryi* Niigata	*Plasmodium falciparum* C10	*Plasmodium chabaudi chabaudi* CB	*Toxoplasma gondii* RH	*Eimeria tenella* Penn State	*Theileria parva* Muguga	*Babesia bovis* T2Bo
Complete/partial	Complete	Partial	Complete	Complete	Complete	Complete	Complete
Size (bp)	34,779	34,682	29,623	34,996	34,750	39,579	33,351
A + T(%)	85.1	86.9	86.3	78.6	79.4	80.5	78.2
IR^z^	+^a^	+	−	+	+	−	−
Protein^b,c^	30	31	31	29	29	32^d^	30^e^
*clpC* ^b^	1	1	1	1	1	2	2
*sufB*	+	+	+	+	+	−	−
*rpl23*	+	+	+	−	−	−	−
*ORF91*	−	+	+	−	−	−	−
rRNA^b^	2^f^	2^f^	2	2^f^	2^f^	2	2
tRNA^b^	25^g^	25^g^	26^h^	24^g^	24^g^	24^i^	24
*trnG* (ACC)	+	+	+	−	−	−	−
Intron in *trnL*(UUA)	+	+	+	+	−	+	+
In-frame stop codon^j^	−	−	−	+	+	−	−
Coding strand	Both	Both	Both	Both	Both	One	One
Accession number	AP013071	X95275, X95276	HF563595	U87145	AY217738	NC_007758	NC_011395

^z^Inverted repeat

^a^ *+* present, *−* absent

^b^Number of different products encoded

^c^ *rpoC2* considered two separate proteins (*rpoC2A* and *rpoC2B*)

^d^Excluding those specified by 12 repetitive genes predicted between *rpoC1* and *rpoC2A*

^e^Excluding repetitive gene products from BBOV_V000300, BBOV_V000310, BBOV_V000320, BBOV_V000180, BBOV_V000200, and BBOV_V000210

^f^The gene for each is present in IR and duplicated

^g^Genes specifying 9 tRNA species (tRNA-Ala(UGC), Arg(ACG), Arg(UCU), Asn(GUU), Ile(GAU), Leu(UAG), Met(CAU), Thr(UGU), and Val(UAC)) are present in IR and duplicated

^h^Two genes encode different versions of tRNA-Thr. The gene for tRNA-Met is duplicated

^i^Five genes encode the same tRNA-Met(CAU)

^j^Excluding the hypothetical codon connecting *rpoC2A* and *rpoC2B*

Each IR unit, which was 5,253 bp, encoded one gene each of large and small ribosomal RNA subunits (*rrl* and *rrs*, respectively) as well as 9 tRNA genes (*trnA*(UGC), *trnI*(GAU), *trnL*(UAG), *trnM*(CAU), *trnN*(GUU), *trnR*(ACG), *trnR*(UCU), *trnT*(UGU), and *trnV*(UAC)), but no protein-coding gene. These characteristics are conserved across all presently studied apicomplexan species with IR-positive apicoplast DNA.

There were 30 total protein-coding genes annotated in the *L. caulleryi* plastid DNA sequence. These genes, as well as 16 tRNA genes, were encoded outside the IR region. Both the content of the genome itself and the arrangement of each gene on the DNA were remarkably highly conserved between *L. caulleryi* and *P. falciparum*. The only difference between the two was the presence of the *ORF91* gene, which is missing from the *L. caulleryi* genome but encoded between *rps5* and *rpl36* (Wilson et al. 1996) in *P. falciparum* DNA. Interestingly, this open reading frame (ORF) is also absent from coccidian and piroplasmid plastid genomes (Table 1), despite the fact that *ORF91* in each *Plasmodium* species specifies a fairly conserved amino acid sequence (data not shown).

**Fig. 2:**
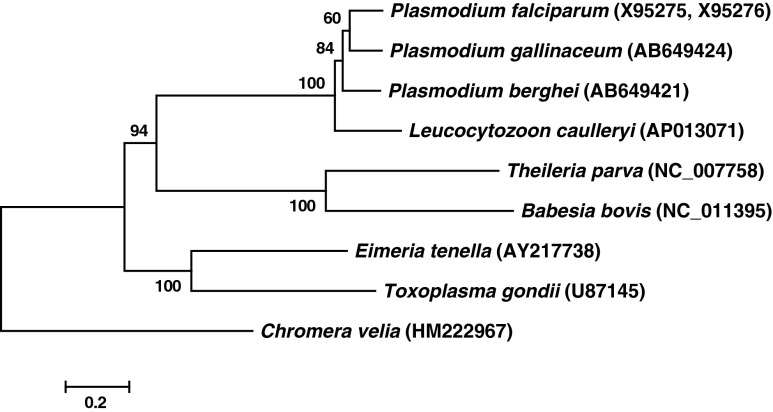
Phylogeny of the apicoplast genomes. Amino acid sequences specified by the 10 selected plastid-encoded genes were concatenated for each species, and the phylogeny was analyzed by the maximum likelihood method with the JTT + F + Γ model to obtain the dendrogram. *Numbers at nodes* indicate bootstrap values from 1,000 replicates. The *scale bar* indicates 0.2 substitutions per site. The accession numbers of the nucleotide sequence data, from which the amino acid sequences used in the analysis were deduced, are given in parentheses after the name of each taxon

Phylogenetic analysis of the concatenated amino acid sequences of 10 gene products (2,478 residues) suggested a close relationship between *L. caulleryi* and *Plasmodium* (Fig. 2). This finding, along with similarities in both gene content and arrangement of genes on the DNA, suggests that the *L. caulleryi* plastid has similar functions to that of *Plasmodium.*

## Discussion

By analogy to *P. falciparum* DNA (Williamson et al. 2002), we had speculated that most of the *L. caulleryi* plastid DNA exists as circular DNA rather than linear multimers, as in *T. gondii* (Williamson et al. 2001). This organization, together with the presence of a set of inverted repeats was confirmed by the sequence analysis.

Phylogenetic studies using apicoplast genome sequences have been used to examine relationships between members of the Apicomplexa. For example, Oborník and colleagues (2002) used 894 nt apicoplast DNA sequences corresponding to part of the *rrs* gene and showed this provide a useful tool to analyze Apicomplexa and distinguish between coccidian and haemosporidian parasites. Our use of concatenated amino acid sequences extends this analysis and suggests a close relationship between *L. caulleryi* and *Plasmodium*.

Both the organization of the genes and the phylogenetic analysis of amino acid sequences suggested that the *L. caulleryi* plastid DNA is very similar to that of *Plasmodium* spp. One notable difference is the absence of *ORF91* from the *L. caulleryi* plastid genome. It is as yet unknown whether the missing gene has been translocated to the nuclear genome so that its protein product is imported into the plastid; however, the absence of *ORF91* in the organellar genome suggests that, unlike *Plasmodium* spp., non-*Plasmodium* species lack the plastid function in which the *ORF91* gene product is involved.

As with other apicoplast DNA, the *rpoC2* gene in *L. caulleryi* specifying a specific subunit of the multi subunit-type DNA-dependent RNA polymerase was interrupted by an in-frame UGA codon. Here we annotated the two juxtaposing ORFs separately as *rpoC2A* and *rpoC2B*, although these might be read through in translation to produce a continuous polypeptide as hypothesized for *rpoC2* in *P. falciparum* (Wilson et al. 1996). Unlike coccidians (http://roos.bio.upenn.edu/∼rooslab/jkissing/toxomap.html, Cai et al. 2003), no other apparent in-frame stop codon has been predicted in the plastid genome of *L. caulleryi*.

The plastid, which is known to provide the platform for critical metabolic processes such as isoprenoid biosynthesis, has been recognized as a promising target for drugs to inhibit the growth of *Plasmodium* spp., and understanding its metabolic functions may aid in designing drugs that prevent the infection and transmission of *L. caulleryi*. Elucidation of its genome structure is an important step along this pathway.

The apicoplast genome of *L. caulleryi* is much larger than the mitochondrial genome of this species (Omori et al. 2008). In the absence of the apicoplast sequence, phylogenetic analysis and genetic typing of *Leucocytozoon* had been generally carried out using mitochondrial sequence such as that of the cytochrome *b* gene alone (Imura et al. 2010; Ortego and Cordero 2009). Now we have established the complete nucleotide sequence of the *Leucocytozoon* apicoplast genome, this newly available information will contribute to improving the accuracy of both phylogenetic analysis and genetic typing of apicomplexan parasites including *Leucocytozoon*.
